# Acknowledgement to Reviewers of *Sports* in 2019

**DOI:** 10.3390/sports8010010

**Published:** 2020-01-20

**Authors:** Sports Editorial Office

**Affiliations:** MDPI, St. Alban-Anlage 66, 4052 Basel, Switzerland

The editorial team greatly appreciates the reviewers who have dedicated their considerable time and expertise to the journal’s rigorous editorial process over the past 12 months, regardless of whether the papers are finally published or not. In 2019, a total of 240 papers were published in the journal, with a median time to first decision of 15 days and a median time from submission to publication of 38.5 days. The editors would like to express their sincere gratitude to the following reviewers for their generous contribution in 2019:
Aadland, EivindGrazer, JacobOlcina, GuillermoAbouhossein, AlirezaGu, WentingOlewnik, Łukasz HubertAglago, Elom KouassiviGuglielmino, ClaudiaOliva, FrancescoAguilar, Miguel PicGutiérrez-García, CarlosOliver, JonAhamed, NizamHa, Min-SeongOlson, Michael W.Almeida Marinho, DanielHaapala, EeroOnofrei, Roxana RamonaAlmeida, Carlos HumbertoHackett, DanielOxford, SamuelAlmeida, Pedro L.Haff, GregPanek-Shirley, Leah M.Alves, Ana Sofia RuivoHandcock, Phil J.Pardos Mainer, ElenaAmin, Nirav H.Hansen, Kjetil FalkenbergParnell, JillAmorim, FabianoHanson, Nicholas J.Parsonage, JoannaAncillao, AndreaHarnish, ChristopherPastor Vicedo, Juan CarlosAnderson, MichaelHassan, Amr Abdulfattah HausienPeart, DanielAndrada, Rafael TimonHassan, IbrahimPedrini, LorenzoAndrews, MichaelHatchett, AndrewPence, BrandtAnnibalini, GiosuèHayes, PhilPerroni, FabrizioAquino, RodrigoHayhurst, JillPethick, JamieArbo, IngeridHayward, Richard D.Petrovic, KimberlyArcher, DavidHealy, RobinPiacentini, Maria FrancescaArdigò, Luca PaoloHeazlewood, IanPic Aguilar, MiguelAssanelli, DeodatoHébert-Losier, KimPiknova, BarboraAtkins, RahshidaHeinrich, KatiePoczta, JoannaAziz, Abdul RashidHernández-García, RaquelPonce-González, Jesús GustavoBabault, NicolasHerold, FabianPosada-Quintero, HugoBabic, VesnaHewson, DavidPraça, GibsonBackes, ToddHodges, LynettePrieto Ayuso, AlejandroBadau, AdelaHolsinger, EvaPrzednowek, KrzysztofBadau, DanaHolvik, KristinPrzybyłowicz, KatarzynaBadicu, GeorgianHornikel, BjoernQuattrocelli, MattiaBalague, NataliaHorsnby, WilliamRama, LuísBallmann, ChristopherHotfiel, ThiloRamirez-Velez, RobinsonBarley, OliverHuang, XiaohuRamos, DomingoBarquín, Roberto RuizIglesias-Soler, EliseoRauter, SamoBaumgart, ChristianIhasz, FerencRaya-González, JavierBeames, SimonIssurin, Vladimir B.Raz, VeredBeretta-Piccoli, MatteoIto, NaokiRen, QingzhongBernstein, Eve R.Iyer, ChitraRibeiro, JoãoBezerra, PedroJaecques, SiegfriedRist, BillymoBhattacharya, AnannyaJames, LachlanRivera, TaniaBinnie, Martyn J.Jee, Yong-SeokRobinson, EdwardBogdanis, GregoryJelleyman, CharlotteRocha-Rodrigues, SílviaBoguszewski, DariuszJensen, BrockRodriguez-Sanchez, NidiaBotek, MichalJochymczyk-Woźniak, KatarzynaRomann, MichaelBotonis, PetrosJohnson, Kelly E.Romeas, ThomasBoussios, StergiosJones, MargaretRomero Martínez, ÁngelBraakhuis, AndreaJówko, EwaRoscoe, ClareBrazo-Sayavera, JavierJoyce, ChrisRumney, PeterBrocherie, FranckJozwiak, MathieuRussell, MarkBrown, LaVerne L.Kanamori, MasaoRutkauskaite, RenataBrumitt, JasonKato, HiroyukiRutkauskas, SauliusBrusseau, Timothy A.Kaviani, MojtabaSaavedra, MiguelBrzeziński, MichałKellis, EleftheriosSacrey, Lori-Ann R.Buckner, SamualKemper, HanSæther, Stig ArveCabri, JanKennedy, RodneySainsbury-Salis, AmandaCamic, Clayton L.Kephart, WesleySánchez, Guillermo Felipe LópezCamplain, RickyKetcham, Caroline J.Sánchez-García, MarioCancela Carral, José MªKhullar, SachinSanchez-Oliver, Antonio JesúsCarballeira, EduardoKim, EungdoSantos, LuisCarper, MichaelKim, Hyun-DuckSarmento, HugoCarroll, Kevin M.Kindy, MarkScheid, Jennifer L.Carson, FraserKingsley, J. DerekSchmitz, BorisCastillo, DanielKingsley, MichaelSchubert, MattCastro-Sanchez, ManuelKirk, BenSchultz, AdrianCastro-Sepulveda, MauricioKlein, MarkusSchwarz, NeilChander, HarishKlemp, AlexSeco, JesusCharmas, MalgorzataKnechtle, BeatSegatto, MarcoChen, YimingKnight, PeterSeiler, StephenChilibeck, PhilKoehler, KarstenSekulic, DamirChing, Congo Tak ShingKonarski, JanSenchina, DavidChoisne, JulieKonefał, MarekSetuain, IgorCholewa, Jason MichaelKorivi, MallikharjunSeverin, Anna CeciliaClarke, NeilKostrzewa-Nowak, DorotaShalfawi, Shaher A. I.Claudino, JoãoKothgassner, Oswald D.Silva, Ana FilipaClemente-Suárez, Vicente JavierKrólikowska, AleksandraSilva, BrunoCollado, Pilar SánchezKump, DavidSilver, TobinCombet, EmilieKwak, Yi-SubSimenko, JozefComfort, PaulLambert, G. PatrickSingh, ChanpreetConte, DanieleLane, AndrewSmeuninx, BenoitCook, MatthewLane, Michael TimothySolano, Pedro RuizCooke, Matthew B.Langeard, AntoineSole, ChristopherCools, WouterLanghammer, BirgittaSousa, AntónioCoratella, GiuseppeLaurin, JérômeSousa, FilipaCortis, CristinaLavender, AndrewSpielmanns, MarcCourel-Ibanez, JavierLeischik, RomanSponsiello, NicolaCouto, BrunoLeón Guereño, PatxiStamatis, AndreasCoutts, Rosanne A.Li, RuiStastny, PetrCropper, EmmaLiedke, GunnarStavrou, VasileiosCuadrado-Peñafiel, VíctorLinthorne, Nicholas P.Stefani, AmbraCynarski, Wojciech J.Lisinskienė, AušraStefani, LauraDadelo, StanislavasLiu, Pei-YangStein, Jesse A.Dalen, TerjeLockard, BrittanieStone, MichaelDames, Kevin D.Lockie, RobertStrazza, AnnachiaraDe La Vega Marcos, RicardoLoi, NatashaStupka, NicoleDe Souza, Eduardo OliveiraLopez Elvira, Jose LuisSuchomel, TimDegens, HansLopez-Fernandez, JorgeSuchomel, Timothy J.Del Vecchio, AlessandroLópez-Gutiérrez, Carlos JavierSuzuki, KatsuhikoDeschodt-Arsac, VeroniqueLópez-López, DanielSzczepek, Agnieszka J.Dettweiler, UlrichLorenzetti, SilvioTaber, ChristopherDoering, TomLorimer, Anna V.Tadeu, PedroDomínguez, RaulLouis, JulienTalpey, ScottDomizio, Marco DiLow, DanielTalpey, Scott W.Donchin, MilkaLuigi Invernizzi, PietroTettenborn, BarbaraDonne, BernardLuis Ubago, JoseThomas, EwanDonti, OlyviaLuk, Hui-YingThompson, RaymondDresp-Langley, BirgittaLuz, CarlosTieland, MichaelDriss, TarakLynn, AnthonyTiidus, PeterD'Souza, Randall FelixLystad, Reidar P.Tomczak, AndrzejDuranti, GuglielmoMack, Diane E.Topa, GabrielaEarnest, ConradMadzima, Takudzwa A.Torres-Luque, GemaEdmonds, RohanMainwaring, Lynda M.Toubekis, ArgyrisEggleston, Jeffrey D.Malchrowicz-Mośko, EwaTrue, LarissaEl Ghoch, MarwanMamen, AsgeirTsekoura, MariaEmerenziani, Gian PietroMandigout, StéphaneTsukamoto, HayatoEmerson, Dawn M.Mann, BryanUeberschär, OlafEstevan, IsaacMarino, JosephVaczi, MarkFalk Neto, Joao HenriqueMarocolo, MoacirValdivia-Moral, PedroFarrell, John W.Martini, DougVan De Heijning, Bert J. M.Federolf, PeterMascherini, GabrieleVan Der Laarse, Willem J.Fernandes, John F. T.Mason, LauraVan Vliet, StephanFernandes, Ricardo J.Mastorci, FrancescaVanDusseldorp, TrishaFernández-Revelles, Andrés B.Maxwell, HazelVaughan, RogerFeuerhake, UdoMayo, XianVentura, JessicaFontana, MarioMcLean, ScottVisser, BartFormenti, DamianoMcLeod, Tamara ValovichVliet, Stephan VanForsyth, AdrienneMcLester, Cherilyn N.Wagle, JohnFoskett, AndrewMedic, NickWagle, John P.Fragoso, Isabel JanuárioMeng, YuWaller, MichaelFrench, DuncanMengarelli, AlessandroWalsh, MarkFulford, JonathanMercer, John A.Waśkiewicz, ZbigniewFuller, ScottMermier, Christine MWawrzyniak, AgataFurness, JamesMethenitis, SpyridonWeakley, JohnathanGaffney, ChristopherMielgo, JuanWeems, MaryGalanis, NikiforosMielgo-Ayuso, JuanWerner, NachbauerGallotta, Maria ChiaraMitschke, ChristianWest, JuliaGarcía-García, OscarMizuguchi, SatoshiWhite, JasonGarcía-Ramos, AmadorMoghadam, Gita KhaliliWillems, MarkGareth, NyeMoir, Gavin L.Williams, RebeccaGąsior, Jakub S.Molero, Pilar PuertasWilson, PatrickGastaldi, LauraMonteiro, DiogoWinchester, LeeGehlert, SebastianMoon, Richard E.Won, DoyeonGentles, JeremyMorales-Suárez-Varela, María M.Wong, AlexeiGepner, YftachMorgan, HaniWoodfield, LorayneGerdin, GöranMorouço, PedroWu, Pei-FungGiannaki, ChristoforosMoser, OthmarWyver, ShirleyGibson, Ann L.Most, JasperYamanashi, HirotomoGil-Calvo, MarinaMousavi, AminYamatsu, KojiGist, NicholasMoya, ManuelZacca, RodrigoGobbi, EricaMüller, PatrickZając, AdamGomes, Thayse NatachaMuñoz, AnaZaras, NikolaosGómez Piqueras, PedroMusumeci, GiuseppeŻebrowska, AleksandraGómez-Baya, DiegoMutz, MichaelZelei, AmbrusGómez-López, ManuelNakashima, MotomuZemková, ErikaGómez-Ruano, Miguel ÁngelNaughton, RobertZenic, NatasaGoncu-Berk, GozdeNavarro, RaulZerbini, GiuliaGonzález Valero, GabrielNeiva, Henrique P.Zhou, ShiGonzález-Badillo, Juan JoséNeville, JonoZinn, CarynGonzález-García, HiginioNg, KwokZinner, ChristophGonzález-Víllora, SixtoNicolas, GuillaumeZouhal, HassaneGoodin, JacobNiederseer, DavidZubac, DamirGorissen, StefanNikolaidis, Pantelis T.Zweiker, DavidGoto, KatsumasaNimphius, SophiaZwingenberger, StefanGoulet, EricNitzsche, Nico
Grabovac, IgorOlaru, Octavian Tudorel


